# HSV-1 reactivation is associated with an increased risk of mortality and pneumonia in critically ill COVID-19 patients

**DOI:** 10.1186/s13054-021-03843-8

**Published:** 2021-12-06

**Authors:** Antoine Meyer, Niccolò Buetti, Nadhira Houhou-Fidouh, Juliette Patrier, Moustafa Abdel-Nabey, Pierre Jaquet, Simona Presente, Tiphaine Girard, Faiza Sayagh, Stephane Ruckly, Paul-Henri Wicky, Etienne de Montmollin, Lila Bouadma, Romain Sonneville, Diane Descamps, Jean-Francois Timsit

**Affiliations:** 1 INSERM, IAME, University of Paris, 75006 Paris, France; 2 Medical and Infectious Diseases Intensive Care Unit, APHP, Bichat University Hospital, Paris, France; 3 Infection Control Program and WHO Collaborating Centre on Patient Safety, Service PCI, University of Geneva Hospitals and Faculty of Medicine, Rue Gabrielle-Perret-Gentil 4, 1205 Geneva, Switzerland; 4 INSERM, UMR1148, Université de Paris, 75018 Paris, France; 5 Virology Department, GH APHP.Nord, Bichat-Claude Bernard University Hospital, 75018 Paris, France; 6 Virology Department, GH APHP.Nord, Université de Paris, IAME INSERM UMR1137, Bichat-Claude Bernard University Hospital, 75018 Paris, France

**Keywords:** HSV, Critically ill, Mortality, COVID-19, Pneumonia, Bacteremia

## Abstract

**Background:**

Data in the literature about HSV reactivation in COVID-19 patients are scarce, and the association between HSV-1 reactivation and mortality remains to be determined. Our objectives were to evaluate the impact of *Herpes simplex virus* (HSV) reactivation in patients with severe SARS-CoV-2 infections primarily on mortality, and secondarily on hospital-acquired pneumonia/ventilator-associated pneumonia (HAP/VAP) and intensive care unit-bloodstream infection (ICU-BSI).

**Methods:**

We conducted an observational study using prospectively collected data and HSV-1 blood and respiratory samples from all critically ill COVID-19 patients in a large reference center who underwent HSV tests. Using multivariable Cox and cause-specific (cs) models, we investigated the association between HSV reactivation and mortality or healthcare-associated infections.

**Results:**

Of the 153 COVID-19 patients admitted for ≥ 48 h from Feb-2020 to Feb-2021, 40/153 (26.1%) patients had confirmed HSV-1 reactivation (19/61 (31.1%) with HSV-positive respiratory samples, and 36/146 (24.7%) with HSV-positive blood samples. Day-60 mortality was higher in patients with HSV-1 reactivation (57.5%) *versus* without (33.6%, *p* = 0.001). After adjustment for mortality risk factors, HSV-1 reactivation was associated with an increased mortality risk (hazard risk [HR] 2.05; 95% CI 1.16–3.62; *p* = 0.01). HAP/VAP occurred in 67/153 (43.8%) and ICU-BSI in 42/153 (27.5%) patients. In patients with HSV-1 reactivation, multivariable cause-specific models showed an increased risk of HAP/VAP (csHR 2.38, 95% CI 1.06–5.39, *p* = 0.037), but not of ICU-BSI.

**Conclusions:**

HSV-1 reactivation in critically ill COVID-19 patients was associated with an increased risk of day-60 mortality and HAP/VAP.

**Supplementary Information:**

The online version contains supplementary material available at 10.1186/s13054-021-03843-8.

## Background

Severe SARS-CoV-2 infection is a life-threatening disease associated with 10–50% mortality [1–8] responsible of respiratory failure and Acute Respiratory Distress Syndrome (ARDS), requiring oxygen support in intensive care unit (ICU). It is associated with immune alterations with profound lymphopenia and requires corticosteroid therapy [2, 9, 10] and immune modulators [11–15] which could affect the risk of superinfections [16]. Hospital-acquired pneumonia (HAP) and particularly ventilator-associated pneumonia (VAP) are frequent among COVID-19 patients, with rates ranging from 29 to 79% [6, 16–21].

Herpes simplex virus (HSV) reactivations have been already described and are common in critically ill patients with ARDS [22–24]. An oropharyngeal HSV reactivation was observed in 20 to 54% of critically ill patients [25–27], and HSV was detectable in lower respiratory tract of up to 64% of mechanically-ventilated patients [27, 28].

Several authors described an impact of HSV tracheobronchitis or pneumonia on mortality [29–31]. Moreover, HSV detection in lower respiratory tract and true HSV bronchopneumonitis were associated with poorer outcomes [27, 32]. Data on the association between HAP/VAP and HSV reactivation are scarce [27, 29]. In March 2020, during the first COVID-19 wave [8], we observed blood and respiratory samples positive for HSV in several patients. Therefore, we decided to systematically monitor on a weekly basis HSV reactivation in blood of critically ill COVID-19 patients admitted to our ICU. Furthermore, each on-demand respiratory samples was also processed for HSV detection.

Our primary objective was to describe patients with HSV reactivation and to evaluate its impact on mortality in critically ill COVID-19 patients. Our secondary objective was to investigate the impact of HSV reactivation on severe healthcare-associated ICU-infections (*i.e.,* HAP/VAP and ICU-acquired bloodstream infections [ICU-BSI]) in COVID-19 patients.

## Materials and methods

### Study design and population

We conducted an observational study using prospectively collected data from patients hospitalized at the Bichat university hospital, a large reference center for COVID-19 patients in the Northern Paris Region.

We included all COVID-19 patients who underwent HSV tests in blood and lower respiratory tract (*i.e.*, in mechanically-ventilated patients) samples on a regular basis.

### Data collection

The study used data from the French prospectively collected multicenter OutcomeRea® database. In accordance with French law, the OutcomeRea® database was approved by the French Advisory Committee for Data Processing in Health Research and the French National Commission for Data Protection and Liberties (registration number 8999262). The database protocol was approved by the Institutional Review Board of the Clermont-Ferrand University Hospital, France. As the research objective of this study was not detailed in the original database protocol, this particular data analysis was then approved by the ethical committee of the Society of Resuscitation of French Language (SRLF), referenced as CE SRLF 21–83. Data storage and data analysis followed MR003 or MR004 methodology as appropriate and had been approved by French legal authorities. Informed consent was not required since the study did not modify patient management, and data were anonymously processed. Oral information was given to the patients or closest family about the possible use of data entered for research purposes, especially about determinants of prognostic of ICU patients.

Demographic, clinical and microbiological data on Polymerase Chain Reaction (PCR)-tested COVID-19 patients were prospectively collected at ICU admission and daily by clinicians.

The following variables at ICU admission were included: demographics, chronic disease/comorbidities, Sequential Organ Failure Assessment (SOFA) scores, body temperature, blood levels of C-Reactive Protein (CRP), D-Dimers, Lactate Dehydrogenase (LDH), and ferritin, ventilation status, and use of immune modulatory agents. Clinical and biological parameters were recorded daily. We also recorded daily data on antiviral agents (including acyclovir, ganciclovir, valganciclovir and valaciclovir) and immunomodulator (corticosteroids, Interleukin-6 receptor antagonists [IL6-ra] and interleukin-1 receptor antagonists [IL1-ra]) medications.

Patients were monitored daily during the ICU stay and followed after ICU discharge until day 60 after admission.

### Study procedures

Blood HSV specimens were prospectively collected on ICU admission and weekly. All mechanically-ventilated COVID-19 patients underwent regularly lower respiratory tract samples for HSV test until removal of the endotracheal tube or death. Endotracheal aspirates, bronchoalveolar fluid, and samples through plugged telescoping catheter were all considered as lower respiratory tract samples. HSV-1 and HSV-2 were routinely screened. All specimens were sent to an accredited reference virology laboratory. Blood and lower respiratory HSV were detected by an automated real-time PCR technology “Simplexa® direct assay HSV1/HSV2 Diasorin®”. HSV-1 and HSV-2 DNA were detected by real-time PCR targeting the HSV polymerase genes. Results for HSV-1 and HSV-2 were provided as positive or negative with PCR cycle threshold (Ct) values.

### Definitions and outcomes

HSV reactivation was defined as a HSV-positive PCR in blood or respiratory samples.

The primary outcome was the 60-day mortality. Secondary outcomes were HAP/VAP and ICU-BSI. HAP was defined as a pneumonia occurring in patients hospitalized for at least 48 h. VAP was defined as a pneumonia occurring in ICU patients mechanically ventilated for at least 48 h [33]. The following three criteria had to be fulfilled: (i) new or progressive and persistent infiltrates or consolidation or cavitation; (ii) pathological body temperature (< 36 °C or > 38 °C) or white blood cell count < 4 or > 12 × 10^3^ cells/mm^3^; (iii) either the new onset/increase of purulent aspirates or worsening gas exchange [33–35]. Endotracheal aspirates, bronchoalveolar fluid, and plugged telescoping catheter samples with semi-quantitative cultures were assessed for microbiological diagnostic with positivity thresholds of 10^6^, 10^4^ and 10^3^ cfu/mL, respectively.

ICU-BSI was defined as a bacteremic episode occurring > 48 h after ICU admission. Typical skin contaminants (*i.e.*, coagulase-negative staphylococci [CoNS]) were included only if ≥ 2 blood cultures showed the same phenotype on separate samples drawn within a 48-h period, or ≥ 1 blood culture positive for clinical sepsis, without any other infectious process, and with an antibacterial agent just initiated by the attending physician [36].

### Statistical analysis

Characteristics of patients were described as median (interquartile range [IQR]) or count (percent) for qualitative and quantitative variables, respectively. HSV positive and negative patients were compared with Chi-square, Fisher and Wilcoxon tests, as appropriate.

The association between HSV-1 reactivation and mortality was estimated using univariable and multivariable Cox models. The variable of interest (HSV-1 reactivation) was forced as a time-dependent variable in the multivariable model. The following covariates were included in the models: age, chronic disease, SOFA score, type of ventilation, use of corticosteroids (*i.e.*, well-known mortality risk factors in critically ill COVID-19 patients). To avoid overfitting with mechanical ventilation variables, only extra-respiratory components of SOFA score were taken into account in the multivariate model. Then, a hazard ratio (HR) for HSV-1 reactivation was derived; a HR > 1 indicated an increased risk for HSV-1 reactivation. The follow-up lasted 60 days. We performed subgroup analyses for blood and respiratory HSV-1 reactivation, respectively. Moreover, we tested the impact of acyclovir therapy on day-60 mortality by sorting HSV-1 positive patients into two groups according to acyclovir treatment.

To evaluate the effect of HSV-1 reactivation on healthcare-associated infections (*i.e.*, HAP/VAP and ICU-BSI), we used cause-specific hazards models. Time zero (T0) was the ICU admission. To determine the risk of healthcare-associated infections, the basis assumption was that censoring was not associated with an altered chance of the event occurring at any given moment. In the current analysis, ICU-mortality was considered as competing event with HAP/VAP or ICU-BSI. The risk of HSV-1 reactivation (*versus* non-HSV-1) as a time-dependent variable was then estimated using Cox cause-specific hazards models. Risk of HAP/VAP or ICU-BSI was expressed in cause-specific hazard ratios (csHR). We performed subgroup analysis for blood and respiratory HSV-1 reactivations, respectively. *p*-values < 0.05 were considered to be significant. Statistical analyses were performed using SAS 9.4 (Cary, North Carolina, USA).

## Results

### Study population

**Fig. 1:**
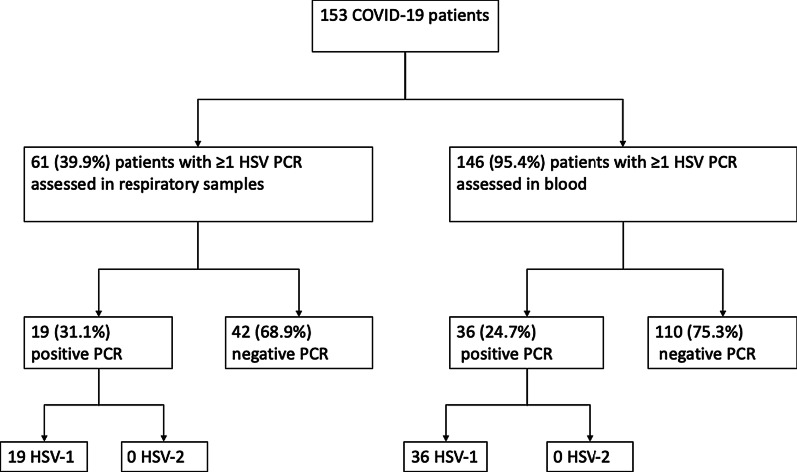
Flowchart. *HSV* Herpes simplex virus. *PCR* polymerase chain reaction

Of the 153 SARS-CoV2 patients admitted for more than 48 h from February 2020 to February 2021 and who underwent at least one HSV screening, respiratory and blood samples were tested from 61/153 (39.9%) and 146/153 (95.4%) patients, respectively (Fig. 1). HSV results were positive in respiratory samples for 19/61 (31.1%) patients and blood samples for 36/146 (24.7%) patients. Fifteen of 153 (9.8%) patients had a HSV-1 reactivation both in blood and respiratory tract: HSV-1 reactivation occurred first in blood for 7/15, in respiratory tract for 6/15 and simultaneously for 2/15 patients.

The median time from ICU admission to the first HSV-positive sample was 14 days (IQR 9.5–18). Importantly, HSV-1 was recovered from all HSV-positive specimens, no reactivation with HSV-2 was observed. Overall, 40/153 (26.1%) patients had at least one positive sample with HSV-1. In total, 481 samples were collected, including 120 respiratory samples: 91 (81.3%) from bronchoalveolar lavage, the other ones from upper tracheal secretions.

### Baseline characteristics

The median age was 60.8 (IQR 50–70) years (Table 1). Patients were mostly men (*n* = 115, 75.2%) and seventy-three (47.7%) had at least one chronic disease; cardiovascular disease and obesity were the most frequently concomitant medical conditions, in 49 (32%) and 62 (40.5%) patients, respectively. The median SOFA score on admission was 5 (IQR 3–7). During the first two ICU days, 52 (33.4%) patients required mechanical ventilation, corticosteroids were administered to 108 (70.6%) patients and tocilizumab or anakinra to 31 (20.3%) patients.

**Table 1:** Baseline characteristics of patients with and without HSV reactivations

		All patients (*n* = 153)	Without HSV reactivation (*n* = 113)	HSV reactivation (*n* = 40)	*p*-value
Female	Number (%)	38 (24.8)	28 (24.8)	10 (25)	1.000
Age	Median (IQR)	60.8 [50.2; 69.5]	60.8 [50.1; 68.8]	61.9 [50.9; 70.8]	0.546
BMI	Median (IQR)	28.8 [25.3; 33]	29 [25.4; 33]	27.7 [24.4; 33.2]	0.629
Diabetes	Number (%)	48 (31.4)	40 (35.4)	8 (20)	0.078
Other chronic diseases*	Number (%)	73 (47.7)	55 (48.7)	18 (45)	0.716
SOFA score	Median (IQR)	5 [3; 7]	5 [3; 7]	5 [4; 7]	0.468
Maximal body temperature (°C)	Median (IQR)	38.2 [37.5; 39.1]	38 [37.5; 39]	38.9 [37.8; 39.3]	0.029
Mechanical ventilation					0.009
None	Number (%)	16 (10.5)	14 (12.4)	2 (5)	
CPAP/HFNO	Number (%)	85 (55.6)	69 (61.1)	16 (40)	
IMV/PEEP < 10 mmHg	Number (%)	0	0	0	
IMV/PEEP > 10 mmHg	Number (%)	40 (26.1)	22 (19.5)	18 (45)	
ECMO	Number (%)	12 (7.8)	8 (7.1)	4 (10)	
Lymphocytes count (/mm3)	Median (IQR)	885 [595; 1220]	890 [610; 1230]	880 [570; 1210]	0.784
Initial use of corticosteroid	Number (%)	108 (70.6)	86 (76.1)	22 (55)	0.016
Tocilizumab/anakinra	Number (%)	31 (20.3)	25 (22.1)	6 (15)	0.492
CRP (mg/L)	Median (IQR)	138.5 [77.5; 204.5]	125 [64.5; 183.5]	180.5 [115.5; 252.5]	0.001
D-Dimers (mg/mL)	Median (IQR)	962 [588; 2043]	952 [564; 1768]	1056.5 [682; 4376]	0.195
Ferritin (ng/mL)	Median (IQR)	1159 [592; 1979]	1060 [526; 1915]	1348 [832; 2370]	0.110
LDH (UI/L)	Median (IQR)	438.5 [337; 606.9]	417 [331; 538]	504.9 [388; 695]	0.019

HSV-positive and -negative patients were compared with Chi-square, Fisher and Wilcoxon tests IQR Inter Quartile Range. These data were collected on ICU admission (day one or two)

*HSV* Herpes simplex virus; *BMI* Body Mass Index; *SOFA* Sequential Organ Failure Assessment; *CPAP* Continuous Positive Airway Pressure; *HFNO* High-Flow Nasal Oxygenotherapy; *IMV* Invasive Mechanical Ventilation; *PEEP* Positive End Expiratory Pressure; *ECMO* Extra-Corporeal Membrane Oxygenation; *CRP* C Reactive Protein; *LDH* Lactate Dehydrogenase

^*^Other chronic diseases were hepatic, heart, respiratory, renal or hematologic diseases, according to the Knaus definitions

HSV-positive and -negative patients were compared with Chi-square, Fisher and Wilcoxon tests IQR Inter Quartile Range. These data were collected on ICU admission (day one or two)

*HSV* Herpes simplex virus; *BMI* Body Mass Index; *SOFA* Sequential Organ Failure Assessment; *CPAP* Continuous Positive Airway Pressure; *HFNO* High-Flow Nasal Oxygenotherapy; *IMV* Invasive Mechanical Ventilation; *PEEP* Positive End Expiratory Pressure; *ECMO* Extra-Corporeal Membrane Oxygenation; *CRP* C Reactive Protein; *LDH* Lactate Dehydrogenase

^*^Other chronic diseases were hepatic, heart, respiratory, renal or hematologic diseases, according to the Knaus definitions

At admission, patients with HSV-1 reactivation during the hospitalization had higher body temperature (38.9 °C *versus* 38 °C; *p* = 0.001), higher CRP levels (180.5 mg/L *versus* 125 mg/L; *p* = 0.001) and higher LDH levels (504.9 UI/L *versus* 417 U/L, *p* = 0.02) than patients without HSV-1 reactivation. The median HSV Ct value of the first positive blood sample was 33.5 (IQR 31.2–35.6), whereas it was 28.2 (IQR 21.4; 33.4) for the first positive respiratory sample.

### HSV-1 reactivation and mortality

Primary and secondary outcomes are described in Table 2. The median length of ICU stay was 23 (IQR 17–40) days and 9 (IQR 6–15) days for patients with and without HSV-1 reactivation (*p* = 0.001), respectively. Eighty-nine patients (58.2%) required invasive mechanical ventilation or Extra-Corporeal Membrane Oxygenation (ECMO). After ICU discharge, only one clinical HSV-1 infection (skin infection) was detected and treated with acyclovir.

**Table 2:** Outcomes in HSV and non-HSV patients

		All patients (*n* = 153)	Without HSV reactivation (*n* = 113)	HSV reactivation (*n* = 40)	*p*-value
Length of stay ICU	Median (IQR)	11.5 [7; 21.5]	9 [6; 15]	23 [17; 40]	< 0.001
Death in ICU	Number (%)	57 (37.3)	36 (31.9)	21 (52.5)	0.024
Death at day 60	Number (%)	61 (39.9)	38 (33.6)	23 (57.5)	0.014
HAP/VAP	Number (%)	67 (43.8)	34 (30.1)	33 (82.5)	< 0.001
ICU-BSI	Number (%)	38 (24.8)	20 (17.7)	18 (45)	0.001
IMV/ECMO	Number (%)	89 (58.2)	54 (47.8)	35 (87.5)	< 0.001

HSV-positive and -negative patients were compared with Chi-square, Fisher and Wilcoxon tests

*HSV* Herpes simplex virus; *IQR* Interquartile range; *ICU* Intensive Care Unit; *HAP* Hospital-acquired pneumonia; *VAP* Ventilator-associated pneumonia; *ICU-BSI* Intensive care unit bloodstream infection. *IMV* Invasive mechanical ventilation. *ECMO* Extra-Corporeal Membrane Oxygenation

Day-60 mortality in the whole cohort was 39.9%, higher in patients with HSV-1 reactivation (57.5% *versus* 33.6% in patients without HSV-1 reactivation, *p* = 0.001).

**Fig. 2:**
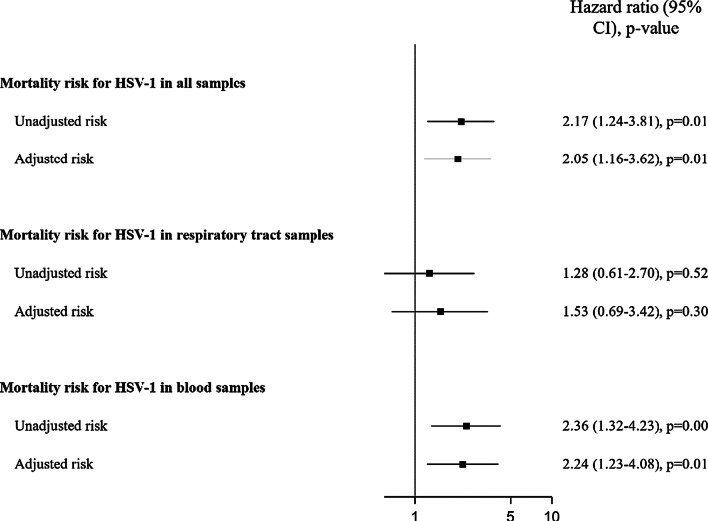
Association between HSV-1 reactivation and mortality at day 60. *HSV* Herpes simplex virus; *SOFA* Sequential Organ Failure Assessment

In univariable Cox models, HSV-1 reactivation was associated with an increased risk of mortality (HR 2.17, 95% CI 1.24–3.81; *p* = 0.007). After adjustment for mortality risk factors (age, oxygenation and ventilation characteristics, extra-respiratory components of SOFA score and corticosteroids therapy), multivariable Cox models showed an increased risk of mortality for HSV-1 reactivation (HR 2.05; 95% CI, 1.16–3.62; *p* = 0.01, Fig. 2, Additional file 1: Table E1).

Among respiratory samples, univariable and multivariable Cox models did not show any association between mortality and HSV-1 reactivation (Fig. 2 and Additional file 1: Table E2).

However, in blood samples, univariable (HR 2.36; 95% CI 1.32–4.23; *p* = 0.004) and multivariable (HR 2.24; 95% CI 1.23–4.08; *p* = 0.009, Fig. 2 and Additional file 1: Table E3) Cox models showed an association between HSV-1 reactivation and mortality.

Among patients with HSV-1 reactivation, acyclovir was administered to twenty-eight (70%) patients, 3 (IQR 2–5) days after positivity in median. After adjustment for mortality factors and using acyclovir as a time-dependent covariate, our Cox model showed an increased risk of mortality at day 60 for patients treated with acyclovir (HR 4.37, 95% CI 2.12–9.02, *p* < 0.001).

### HSV-1 reactivation and healthcare-associated ICU infections

**Fig. 3:**
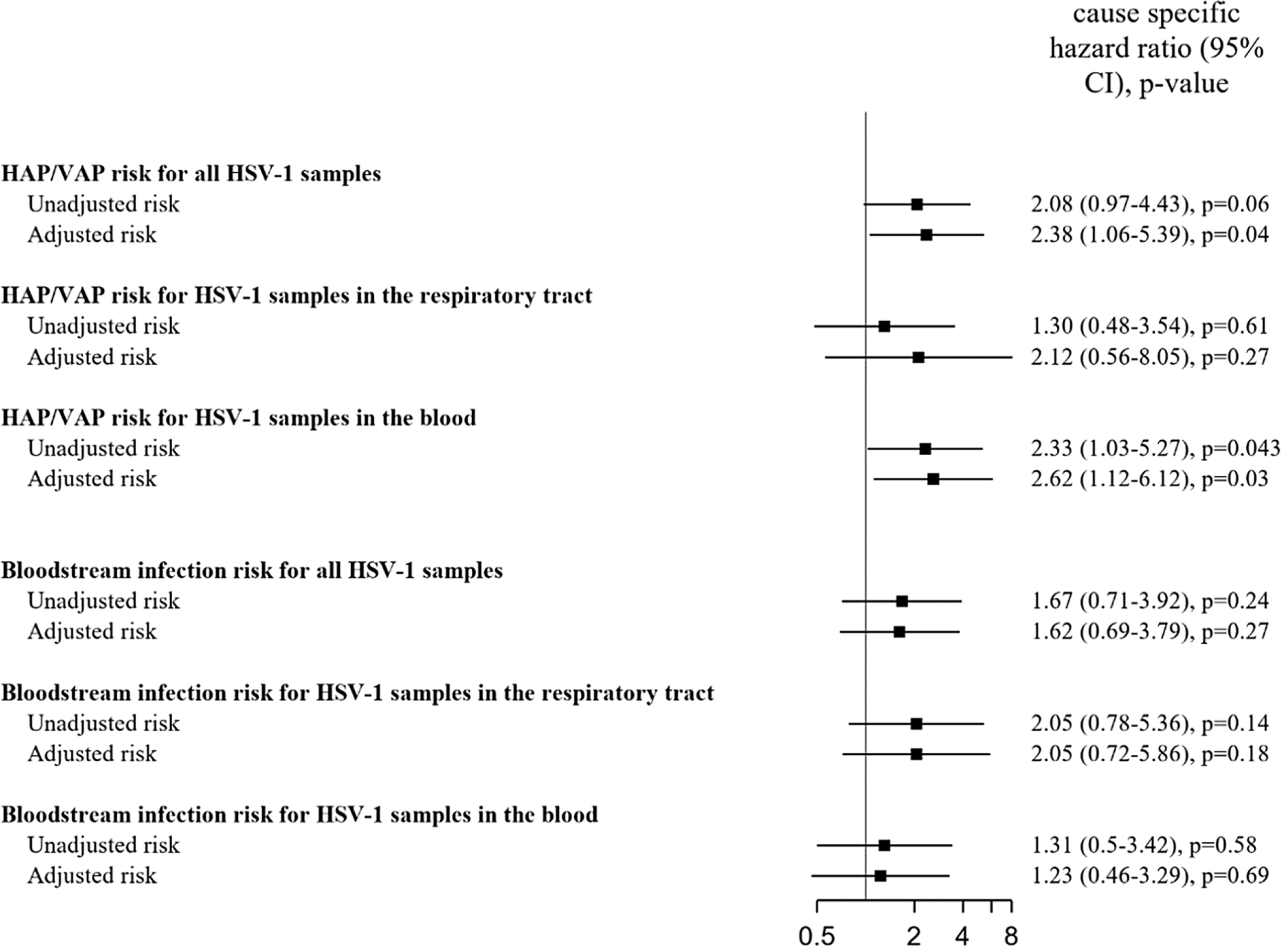
Association between HSV detection and nosocomial infections (hospital acquired pneumonia/ventilator associated pneumonia and bacteremia). NB: Adjustment factors were age, chronic disease, extra-respiratory SOFA score, type of ventilation, use of corticosteroids. To avoid overfitting with mechanical ventilation variables, only extra-respiratory components of SOFA score were taken into account. *SOFA* Sequential Organ Failure Assessment

Sixty-seven (43.8%) patients developed a HAP/VAP. In univariable cause-specific models, HSV-1 reactivation was not significantly associated with an increased risk of HAP/VAP (csHR 2.08, 95% CI 0.97–4.43, *p* = 0.059). Multivariable specific cause models showed an increased risk of HAP/VAP (csHR 2.38, 95% CI 1.06–5.39, *p* = 0.037, Fig. 3 and Additional file 1: Table E4). Among respiratory samples, cause-specific models did not show any association between mortality and HSV-1 reactivation (data not shown). In blood samples, multivariable cause-specific models (HR 2.62; 95% CI 1.12–6.12; *p* = 0.027, Additional file 1: Table E5) showed an association between HSV-1 reactivation and HAP/VAP. We did not find a significant different distribution of pathogens identified in patients with or without HSV-1 reactivation in the respiratory tract (data not shown). We observed two HAPs after ICU discharge.

ICU-BSI occurred in 38 (24.8%) patients, in 18 (45.0%) patients with HSV-1 reactivation *versus* 20 (17.7%) patients without, respectively. Univariable (csHR 1.67; 95% CI 0.71–3.92; *p* = 0.235) and multivariable (csHR 1.62; 95% CI 0.69–3.79; *p* = 0.269) cause-specific models did not show any increased risk for ICU-BSI (Fig. 3). Similar results were observed in the different subgroup analyses.

## Discussion

Using high-quality prospectively collected data from all COVID-19 patients admitted to a large French COVID-19 reference center, we showed that mortality and HAP-VAP risks were increased in patients with HSV-1 reactivation. However, the risk for ICU-BSI was not increased in patients with HSV-1 reactivation. In our cohort none of the COVID-19 patients had HSV-2 reactivation.

Data on HSV reactivation in COVID-19 patients are scarce. Only one small study described HSV reactivation in lower respiratory tract of COVID-19 patients [37] without specifically investigating its impact on mortality or ICU-acquired infections. Furthermore, to our knowledge, no data on HSV detection in blood samples of COVID-19 patients were available. Interestingly, we observed higher rates of HSV positive PCR in the blood compared to previous studies including non-COVID-19 patients [38, 39]. This finding may be explained by immune alterations with profound leucopenia [2, 40] and a large use of immunomodulators in COVID-19 patients [11, 14].

We showed that HSV-1 reactivation in COVID-19 patients was associated with an increased mortality.

Impact of HSV reactivation on prognostic among critically ill non-COVID-19 patients is well known. Several authors showed an increased morbidity and mortality [25, 29, 30, 41]. However, data on HSV-1 reactivation among COVID-19 patients are scant [37]. Le Balch et al.showed that HSV-1 reactivation among severe COVID-19 patients was associated with a longer duration of invasive mechanical ventilation and a longer length of stay, but without any impact on mortality. However, due to the low number of included patients (*n* = 38), adjusting for confounding factors was impossible. To the best of our knowledge, we performed the first study that prospectively investigated HSV-1 reactivation in COVID-19 patients. The relevance of HSV-1 reactivation and its real impact on mortality remains controversial [42]. On the one hand, HSV could be a simple bystander and its presence not reflect a direct pathogenicity [43–45]. Due to the observational nature of our study, we could not determine causality between HSV and mortality since many confounders both related with HSV-1 reactivation and mortality might have been unmeasured. It may be especially the case for immune paralysis that might have induced both HSV-1 reactivation and increased the mortality risk [46–48]. In our study, most patients did not demonstrate reactivation until about two weeks. Patients who had HSV-1 reactivation had higher levels of inflammation at admission, suggesting a different immune response comparing to patients without HSV-1 reactivation. Recently, Seeßle et al. [49] showed an increase in activated CD8 cells followed by a drop in interferon stimulated gene expression occurring after HSV-1 reactivation, also occurring in a similar time frame to what was seen in our study. This suggests some form of immune failure leading to mortality with HSV-1 reactivation just being an epiphenomenon. However, since our analyses were adjusted for severity of critical illness, a direct pathogenic role of HSV may be hypothesized. A recent meta-analysis [50] showed a decreased mortality among HSV patients receiving acyclovir. In light of these considerations, it could be possible that HSV is a true pathogen inducing pulmonary parenchyma damages. However, the only randomized trial evaluating the use of acyclovir in HSV reactivation [28] showed a marginal and non-significant impact on mortality, thus weakening this hypothesis. Of note, our data showed that adequate early acyclovir therapy was not associated with a prognostic improvement of HSV-1 positive patients. Although our study was not designed to evaluate the impact of acyclovir and these data were issued from a complementary analysis, it suggested that HSV-1 reactivation was more a marker of poor immune status than a disease that would need specific therapy. Further studies are necessary to determine if early acyclovir therapy could improve outcomes.

We observed that HSV-1 reactivation was associated with an increased risk of HAP/VAP. Interestingly, only a few studies investigated the association between HSV reactivation and VAP in critically ill patients. Daubin et al. [51] performed a prospective observational study and determined the incidence of VAP among intubated patients in ICU. HSV-1 was recovered in only 31% of VAP patients. To date, the association between HAP/VAP and HSV has not been studied in COVID-19 patients.

We showed an association between HSV-1 reactivation and HAP/VAP which remained significant after adjustment on several factors. On the one hand, our findings suggested that HSV-1 reactivation could induce lung injuries which could promote bacterial superinfections. On the other hand, it is conceivable that lesions induced by superinfections may promote HSV reactivation in lung and/or blood. The causality relationship between HAP/VAP and HSV reactivation remains to be elucidated. Overall, COVID-19 exposes critically ill patients to an increased risk of superinfections and HSV reactivation by induced immunosuppression and severe lung damages. HSV-1 reactivation among COVID-19 patients could help clinicians to identify patients at risk of developing superinfections. Moreover, frequent microbiological samples may be collected in order to early identify HAP/VAP.

Our study has several limitations. First, we performed an observational study and residual confounding cannot be excluded. Therefore, associations between HSV-1 and mortality or HAP/VAP should be interpreted with caution. However, our multivariable models allowed us to adjust for several factors. It may be debatable to do not use IL1-ra or IL6-ra exposition as an adjustment covariate for multivariable Cox Models. Actually, Tocilizumab seems to improve outcomes in recent studies [13, 52]. Nevertheless, only 31 (20.3%) patients received Interleukin-receptor antagonists ([IL-ra] *i.e.,* Anakinra or Tocilizumab) in the first two ICU days in our cohort. The association between IL-ra and mortality using univariables Cox models was not significant (HR 1.04, 95% CI 0.54–2.01, *p* = 0.90). Therefore, we did not include IL-ra as adjustment covariate. Second, HSV reactivation in respiratory tract was tested only in intubated patients, which may result in a selection of patients with poorer prognostic. Nevertheless, HSV reactivation in blood was also tested in non-intubated patients, thus mitigating this selection bias. Moreover, our subgroup analyses showed an increased risk of mortality and HAP/VAP for HSV-1 reactivation in blood, thus suggesting that blood HSV-1 reactivation played a predominant role (*i.e.,* compared to respiratory HSV-1 reactivation). Third, in our main analysis, we pooled respiratory and blood samples, thus assuming that HSV positive blood PCR was mostly due to a pulmonary reactivation. Our analyses showed a predominant relevance of blood HSV reactivation. It may be debatable that a HSV reactivation in blood may be associated with a respiratory HSV reactivation. Fourth, median time to HSV-1 reactivation was shorter than median length of ICU stay for patients without HSV-1 reactivation (14 days *versus* 9 days, respectively). Some patients without HSV-1 reactivation leaved the ICU before HSV-1 reactivation could occur. HSV-1 was not routinely tested after ICU discharge so a selection bias may occur. Nevertheless, even if ICU HSV screening was not performed after ICU discharge, we reviewed all patient courses and we detected only one case with a HSV infection (HSV skin infection): we suppose that event did not substantially modify the results. Moreover, no clinical or biological signs of HSV-1 reactivation were described so we could reasonably assume that no patient had HSV-1 reactivation after ICU discharge. Fifth, our analysis suggested that blood sampling may be sufficient to monitor HSV-1 reactivation. Nevertheless, we have to nuance that notion. We had a potential selection bias caused by the lack of respiratory samples for non-intubated patients which may result in a selection of patients with poorer prognosis. Moreover, our subgroup analysis for HSV-1 reactivation in respiratory tract showed a non-significant trend for healthcare-associated ICU-infections with a HR > 1, particularly for bloodstream infection. Sixth, we performed a monocentric study, thus limiting the generalizability to other ICU settings. However, a large sample size with high quality data was collected. Finally, our findings should be limited to critically ill patients. The rate of respiratory HSV reactivation in non-critically ill patients remained unresolved and further studies are needed in this context.

## Conclusion

Critically ill COVID-19 patients frequently reactivate HSV-1 but not HSV-2. HSV-1 reactivation in critically ill COVID-19 patients was associated with an increased risk of day-60 mortality and HAP/VAP occurrence. The association between HSV-1 reactivation and mortality may be useful for the clinician to identify patients with the worst prognosis. Further studies are necessary to determine the causality relationship.

## Supplementary Information

**Additional file 1.** **Table E1.** Univariable and multivariable Cox models investigating the association between HSV-1 reactivation and mortality at day 60. **Table E2.** Univariable and multivariable Cox models investigating the association between HSV-1 reactivation and mortality at day 60 in respiratory samples. **Table E3.** Univariable and multivariable Cox models investigating the association between HSV-1 reactivation and mortality at day 60 in blood samples. **Table E4.** Univariable and multivariable cause specific models investigating the association between HSV-1 reactivation and HAP/VAP. **Table E5.** Univariable and multivariable cause specific models investigating the association between HSV-1 reactivation in blood and HAP/VAP.

## Data Availability

The datasets used and/or analyzed during the current study are available from the corresponding author on reasonable request.

## Abbreviations

ARDS: Acute respiratory distress syndrome
CoNS: Coagulase-negative staphylococci
CRP: C-reactive protein
cs: Cause-specific
Ct: Cycle threshold
ECMO: Extra-corporeal membrane oxygenation
HAP: Hospital-acquired pneumonia
HR: Hazard risk
HSV: Herpes simplex virus
ICU: Intensive care unit
ICU-BSI: Intensive care unit bloodstream infection
IL-ra: Interleukin receptor antagonists
IL1-ra: Interleukin-1 receptor antagonists
IL6-ra: Interleukin-6 receptor antagonists
IQR: Interquartile range
LDH: Lactate dehydrogenase
PCR: Polymerase chain reaction
SOFA: Sequential organ failure assessment
T0: Time 0
VAP: Ventilator-associated pneumonia
