# Supplementary material for: HSV-1 reactivation is associated with an increased risk of mortality and pneumonia in critically ill COVID-19 patients
Source: Crit Care. 2021 Dec 6;25:417. doi: 10.1186/s13054-021-03843-8 (PMC8647503; doi:10.1186/s13054-021-03843-8)
Supplement: Supplementary file 1 — Additional file 1. Table E1. Univariable and multivariable Cox models investigating the association between HSV-1 reactivation and mortality at day 60. Table E2. Univariable and multivariable Cox models investigating the association between HSV-1 reactivation and mortality at day 60 in respiratory samples. Table E3. Univariable and multivariable Cox models investigating the association between HSV-1 reactivation and mortality at day 60 in blood samples. Table E4. Univariable and multivariable cause specific models investigating the association between HSV-1 reactivation and HAP/VAP. Table E5. Univariable and multivariable cause specific models investigating the association between HSV-1 reactivation in blood and HAP/VAP. [file 13054_2021_3843_MOESM1_ESM.docx]

**Supplemental digital content**

**Table E1. Univariable and multivariable Cox models investigating the association between HSV-1 reactivation and mortality at day 60**

|  | Univariable Cox model | | Multivariable Cox model | |
| --- | --- | --- | --- | --- |
| Parameter | **HR (95% CI)** | **P value** | **HR (95% CI)** | **P value** |
| HSV-1 reactivation | 2.17 (1.24 – 3.81) | 0.007 | 2.05 (1.16 – 3.62) | 0.013 |
| Age |  |  | 1.04 (1.01 – 1.06) | 0.003 |
| Chronic disease |  |  | 1.34 (0.78 – 2.28) | 0.292 |
| Extra respiratory SOFA score |  |  | 1.07 (0.97 – 1.19) | 0.184 |
| Mechanical ventilation  None  CPAP/HFNO  IMV/PEEP < 10 mmHg  IMV/PEEP > 10 mmHg  ECMO |  |  | 1  0.82 (0.28 – 2.38)  0.99 (0.31 – 3.23)  1.26 (0.30 – 5.28) | 0.709  0.991  0.750 |
| Initial use of cortisteroids |  |  | 1.54 (0.87 – 2.74) | 0.139 |

Legend. HR Hazard Ratio; CI Confidence interval; HSV *Herpes simplex virus;* SOFA Sepsis-related Organ Failure Assessment; CPAP Continuous Positive Airway Pressure; HFNO High Flow Nasal Oxygenotherapy; IMV Invasive Mechanical Ventilation; PEEP Positive End Expiratory Pressure; ECMO Extra Corporeal Membrane Oxygenation. NB to avoid overfitting with mechanical ventilation variables, only extra-respiratory components of SOFA score were taken into account.

**Table E2. Univariable and multivariable Cox models investigating the association between HSV-1 reactivation and mortality at day 60 in respiratory samples**

|  | Univariable Cox model | | Multivariable Cox model | |
| --- | --- | --- | --- | --- |
| Parameter | **HR (95% CI)** | **P value** | **HR (95% CI)** | **P value** |
| HSV-1 reactivation | 1.28 (0.61 – 2.69) | 0.519 | 1.53 (0.69 – 3.42) | 0.299 |
| Age |  |  | 1.04 (1.01 – 1.08) | 0.016 |
| Chronic disease |  |  | 1.02 (0.49 – 2.12) | 0.963 |
| Extra respiratory SOFA score |  |  | 1.02 (0.89 – 1.18) | 0.736 |
| Mechanical ventilation  None  CPAP/HFNO  IMV/PEEP < 10  IMV/PEEP > 10  ECMO |  |  | 0.48 (0.10 – 2.34)  0.81 (0.15 – 4.34)  0.86 (0.12 – 6.21) | 0.363  0.809  0.881 |
| Initial use of corticosteroids |  |  | 1.37 (0.64 – 2.94) | 0.423 |

Legend. HR Hazard Ratio; CI Confidence interval; HSV Herpes simplex virus; SOFA Sepsis-related Organ Failure Assessment; CPAP Continuous Positive Airway Pressure; HFNO High Flow Nasal Oxygenotherapy; IMV Invasive Mechanical Ventilation; PEEP Positive End Expiratory Pressure; ECMO Extra Corporeal Membrane Oxygenation. NB to avoid overfitting with mechanical ventilation variables, only extra-respiratory components of SOFA score were taken into account.

**Table E3. Univariable and multivariable Cox models investigating the association between HSV-1 reactivation and mortality at day 60 in blood samples**

|  | Univariable Cox model | | Multivariable Cox model | |
| --- | --- | --- | --- | --- |
| Parameter | **HR (95% CI)** | **P value** | **HR (95% CI)** | **P value** |
| HSV-1 reactivation | 2.36 (1.32 – 4.23) | 0.004 | 2.24 (1.23 – 4.08) | 0.009 |
| Age |  |  | 1.03 (1.00 – 1.05) | 0.024 |
| Chronic disease |  |  | 1.41 (0.80 – 2.49) | 0.236 |
| Extra-respiratory SOFA score |  |  | 1.04 (0.93 – 1.17) | 0.462 |
| Mechanical ventilation  None  CPAP/HFNO  IMV/PEEP < 10  IMV/PEEP > 10  ECMO |  |  | 1  1.12 (0.33 – 3.78)  1.25 (0.34 – 4.63)  1.21 (0.24 – 6.02) | 0.852  0.735  0.819 |
| Initial use of cortisteroids |  |  | 1.53 (0.85 – 2.77) | 0.156 |

Legend. HR Hazard Ratio; CI Confidence interval; HSV *Herpes simplex virus;* SOFA Sepsis-related Organ Failure Assessment; CPAP Continuous Positive Airway Pressure; HFNO High Flow Nasal Oxygenotherapy; IMV Invasive Mechanical Ventilation; PEEP Positive End Expiratory Pressure; ECMO Extra Corporeal Membrane Oxygenation. . NB to avoid overfitting with mechanical ventilation variables, only extra-respiratory components of SOFA score were taken into account.

**Table E4. Univariable and multivariable cause specific models investigating the association between HSV-1 reactivation and HAP/VAP.**

|  | HAP/VAP | | Death | |
| --- | --- | --- | --- | --- |
|  | **csHR (95% CI)** | **P value** | **csHR (95% CI)** | **P value** |
| Univariable model | 2.08 (0.97 – 4.43) | 0.059 | 0.76 (0.15 – 3.78) | 0.740 |
| Multivariable model * | 2.38 (1.06 – 5.39) | 0.037 | 1.15 (0.20 – 6.68) | 0.880 |

Legend. HAP Hospital acquired pneumonia; VAP Ventilator associated pneumonia; csHR cause-specific hazard ratio; CI Confidence interval

*Adjustment factors: age, chronic disease, extra-respiratory SOFA score, type of ventilation, use of corticosteroids

**Table E5. Univariable and multivariable cause specific models investigating the association between HSV-1 reactivation in blood and HAP/VAP**

|  | HAP/VAP | | Death | |
| --- | --- | --- | --- | --- |
|  | **csHR (95% CI)** | **P value** | **csHR (95% CI)** | **P value** |
| Univariable model | 2.33 (1.03 – 5.27) | 0.043 | 0.93 (0.18 – 4.89) | 0.928 |
| Multivariable model * | 2.62 (1.12 – 6.12) | 0.027 | 1.64 (0.27 – 10.1) | 0.595 |

Legend. HAP Hospital acquired pneumonia; VAP Ventilator associated pneumonia; csHR cause-specific hazard ratio; CI Confidence interval

*Adjustment factors: age, chronic disease, extra-respiratory SOFA score, type of ventilation, use of corticosteroids
